# Climate change and body size trends in aquatic and terrestrial endotherms: Does habitat matter?

**DOI:** 10.1371/journal.pone.0183051

**Published:** 2017-08-16

**Authors:** Daniel E. Naya, Hugo Naya, Joseph Cook

**Affiliations:** 1 Departamento de Ecología y Evolución, Facultad de Ciencias, Universidad de la República, Montevideo, Uruguay; 2 Unidad de Bioinformática, Institut Pasteur de Montevideo, Montevideo, Uruguay; 3 Departamento de Producción Animal y Pasturas, Facultad de Agronomía, Universidad de la República, Montevideo, Uruguay; 4 Department of Biology and the Museum of Southwestern Biology, University of New Mexico, Albuquerque, New Mexico, United States of America; University of Sao Paulo, BRAZIL

## Abstract

Several studies have claimed that reduction in body size comprises a nearly universal response to global warming; however, doubts about the validity of this pattern for endothermic species have been raised recently. Accordingly, we assessed temporal changes in body mass for 27 bird and 17 mammal species, to evaluate if a reduction in body size during the 20^th^ century is a widespread phenomenon among endothermic vertebrates. In addition, we tested if there are differences in the temporal change in size between birds and mammals, aquatic and terrestrial species, and the first and second half of the 20^th^ century. Overall, six species increased their body mass, 21 species showed no significant changes in size, and 17 species decreased their body mass during the 20th century. Temporal changes in body mass were similar for birds and mammals, but strongly differ between aquatic and terrestrial species: while most of the aquatic species increased or did not change in body mass, most terrestrial species decreased in size. In addition, we found that, at least in terrestrial birds, the mean value of the correlation between body mass and year of collection differs between the first half and the second half of the 20^th^ century, being close to zero for the former period but negative for the later one. To our knowledge, this is the first study showing that temporal changes in body mass differ between aquatic and terrestrial species in both mammals and birds.

## Introduction

Several studies have claimed that a reduction in body size comprises the third universal ecological response to global warming, after species distributional shifts and phenological changes [1–3]. For the particular case of endothermic species, two non-mutually exclusive ideas are usually invoked to explain this phenomenon [4–7]. First, a rise in ambient temperature may cause a reduction in body size due to energetic considerations –i.e., smaller size implies a larger surface to volume ratio and is “better” for heat dissipation in warmer climates– in line with Karl Bergmann’s famous argument [8]. Second, a decrease in resource availability (or quality) could cause a reduction in the rate of growth throughout an animal’s development, resulting in a smaller adult size. As for the mechanistic basis of these changes, two non-mutually exclusive explanations are usually advanced: a plastic response at the individual level [9–10] and genetic changes at the population level [11–12]. Even though very few studies have evaluated the relative weight of these two mechanisms, current evidence suggests that phenotypic plasticity usually accounts for a higher portion, if not for all, of the overall phenotypic change [13–14].

**Fig 1:**
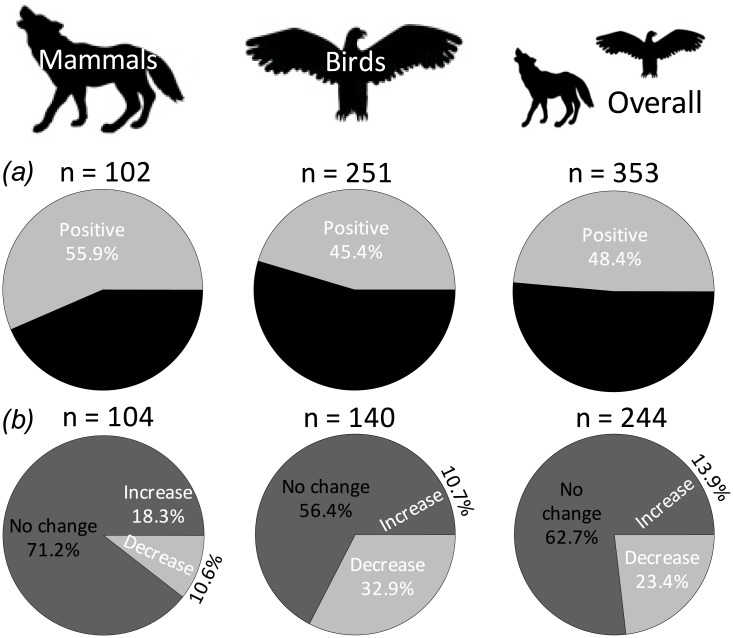
Percentege of bird and mammal populations that show different temporal responses in body size, taking into account *(a)* only the sign of the changes (upper panels), and *(b)* the statistical significance of the changes (lower panels). Data were analyzed separately for trend and significance because statistical significance depends on sample size (which varies from case to case), and also because some studies only report the sign of the temporal trend, while other studies only report that changes were not significant (see S1 Dataset for details).

However, leaving aside the potential causes and the mechanistic basis for the observed changes, doubts have been raised recently about the validity of a relationship between temperature and body-size in endothermic species [5, 15–18]. In line with this, our semi-quantitative review of current evidence on temporal change in body size for endothermic vertebrates suggests that no clear pattern is evident. Specifically, after analyzing data from 46 studies, comprising a total of 369 populations of birds and mammals (S1 Dataset), we conclude that: (i) regardless of the statistical significance of the changes, the proportion of populations that increase their size is similar to the proportion of populations that decrease their size (Fig 1a), and (ii) when the statistical significance of the changes is taken into account, nearly two-thirds of populations analyzed to date showed no significant temporal changes in body size (Fig 1b). In addition, two intriguing points emerge from this semi-quantitative assessment. First, with a couple of exceptions, all the studies analyzing only a single population reported significant temporal change in size, while all the studies analyzing more than one population (or species) indicate that no significant changes are common. Such differences clearly suggest a publication bias that could affect impressions about the ubiquity of temporal changes in body size (see [15]). Second, with very few exceptions, studies analyzing more than one population of the same species, or the combination of different studies evaluating different populations of the same species, indicate that observed responses usually vary, in both the sign of the trend and statistical significance, from one population to the other. That is, there is significant variation in body size changes from site to site, which suggest that idiosyncratic responses, probably linked to differences in local conditions [19] or even to dispersal events between localities [20], are common. In summary, it is clear that we need to evaluate much more data before accepting that a reduction in body mass associated with global warming or, more generally, with global environmental change, comprises a general rule for endotherms. In particular, we need to conduct more multi-species comparative studies, to understand the natural variation in phenotypic responses, and use similar methods (e.g., the same measurement of body size) across large geographic areas, to reduce the incidence of confounding factors and to account for potential site-to-site fluctuations.

Within this context, we assessed temporal change in body mass for 27 bird and 17 mammal species to evaluate if a reduction in body size during the 20^th^ century, whatever its causes and mechanistic basis, is a widespread phenomenon among endothermic vertebrates. In addition, we tested whether there are differences in the temporal response between: (1) birds and mammals, (2) aquatic and terrestrial species, and (3) the first and second half of the 20^th^ century. To the best of our knowledge, this is the first study that: (i) simultaneously assesses temporal changes in body size for birds and mammals, using the same methodology to analyze both taxonomic groups and accounting for the effect of phylogeny, and (ii) evaluates differences in the temporal responses between aquatic and terrestrial species.

## Methods

### Data base description

To assess temporal change in body size, we downloaded data on body mass for endothermic vertebrates, with the exception of rodents and passerine birds due to logistic constraints (i.e., our inability to curate two datasets with several thousands of records) from Arctos, an online natural history database (www.arctos.database.museum). We included all species with more than 100 geo-referenced records of sexed individuals, covering the last four decades at least. We excluded six species for which most of the data (> 80%) span < 10 years of collection (Figure A in S1 Appendix), and removed 26 outlier records corresponding to very small individuals, in which body mass was close to zero. Those selection criteria yielded a total of 20,496 records belonging to 44 species (S2 Dataset). These species were then classified, based on information provided by IUCN (http://www.iucnredlist.org/), Birdlife International (http://www.birdlife.org/), and the Museum of Zoology of the University of Michigan (http://animaldiversity.org/), as aquatic, if they inhabit fresh water and/or marine habitats in addition to terrestrial ones (birds: n = 10, mammals: n = 4), or terrestrial, if they only inhabit terrestrial habitats (birds: n = 17, mammals: n = 13). By analyzing multiple populations of each species over broad geographical areas (see Figure B in S1 Appendix), our analysis intended to identify responses at the species level, rather than local responses at the population level. Also, our analysis assumed that the effects of several factors that could affect body mass and that we cannot filter statistically (e.g., age of each individual, season of the year, time of the day of capture, people taking the measurements) are randomly distributed among years.

### Comparison between taxonomic groups and species habitats

We estimated the partial correlation coefficient between body mass and the year of collection (r_year_) for each species separately, through conventional linear regression models, using (absolute) latitude, longitude, and sex as covariates in the models. Then, we evaluated differences in r_year_ between taxonomic groups (i.e., birds or mammals) and species habitats (i.e., aquatic or terrestrial) using a two-way ANOVA. The correlation coefficient between body mass and the year of collection (r_year_) was not correlated with sample size, species body mass, or species distribution, but was correlated with the length of the time series (r = 0.36, P = 0.02). Hence, in addition to the ANOVA, we also ran an ANCOVA using the length of time series as a covariate. In these analyses, we used the Kolmogorov-Smirnov test to assess normality, Levene’s test to assess homogeneity of variance, and (in ANCOVA) a parallelism test to assess the interaction between factors and the covariate. All these analyses were done with Statistica (version 8.0) software [21], with statistical significance established at the α = 0.05 level.

To take into account phylogenetic relationships among species, the relationship between body mass and the year of collection was also evaluated using a phylogenetic generalized linear mixed model (PGLMM) [22], with species as a random factor, taxonomic group and habitat as fixed factors, and (absolute) latitude, longitude, sex and length of the time series as covariates (model A). To consider potential interactions between the year of collection and fixed factors, we ran two additional models: one including the interaction term with taxonomic group (model B) and the other including the interaction term with habitat (model C). In all these models, species body mass was standardized to a mean equal to zero and variance equal to one. The phylogenetic tree used in these analyses (Figure C in S1 Appendix) was based on previously published papers for birds [23] and mammals [24–25]. Given that branch length is not known for this tree, we set all lengths to one. Phylogenetic analyses were run with the MCMCglmm package [26] in the free software R [27]. Inferences for each regression model were based on 200,000 samples, obtained after discarding 50,000 samples as burn in. In all models, default priors were used for “fixed” effects, while inverse Wishart priors with scale parameter equal to half of dependent variable variance (and 3 degrees of freedom) were used for “random" effects. A thinning interval of 100 was used for computing features of the posterior distribution. Convergence diagnostics, statistical and graphical analyses of Markov chain Monte Carlo (MCMC) sampling output were carried out with the CODA package [28] available in R. Finally, the significance of each regression coefficient was estimated from Markov chain Monte Carlo p-value (pMCMC), which basically represents (2x) the proportion of posterior values that are of the opposite sign to the parameter estimate, whereas the fit of each model to the data was assessed through Deviance Information Criterion (DIC scores).

### Temporal variation in the temporal responses

Because data for terrestrial bird species are fairly evenly distributed in time through most of the 20^th^ century (see Figure B in S1 Appendix), we decided to evaluate temporal changes in the relationship between body mass and year of collection with this group of species. For that purpose, we split the series of each species into two periods of time: one from the earliest date of collection (*ca*. 1915) to 1950, representing a relatively low intensity of change in environmental conditions, and the other from 1951 to the latest date of collection (*ca*. 2013), representing a relatively high intensity of change in environmental conditions [29]. Note that the increase in globally averaged temperature over the period 1901–2012 was about 0.89°C, while the increase over just the period 1951–2012 was about 0.72°C [29]. Thus, the rate of change in ambient temperature for our second period (*ca*. 0.12°C per decade) is almost four times greater than for our first period of time (*ca*. 0.03°C per decade). For each species and period of time, we calculated the partial correlation for the year of collection (r_year_) using (absolute) latitude, longitude and sex as covariates in the regression models. Then, we evaluated difference in the mean value of r_year_ between both periods of time, using a paired *t-*test for dependent samples. These analyses were done with Statistica (version 8.0) software, and statistical significance was established at the α = 0.05 level. Finally, to take into account phylogenetic relationships among species, we estimated the relationship between body mass and the year of collection separately for each period of time (i.e., before and after 1950), using phylogenetic generalized linear mixed models (PGLMM) [22], with (absolute) latitude, longitude, and sex as covariates. Then, the significance of each regression coefficient was estimated from Markov chain Monte Carlo p-value (pMCMC). In addition, as in the Bayesian MCMC context it is possible to directly obtain samples of the regression coefficients from the posterior distribution for each period of time, we were able to generate a new distribution from the differences between regression coefficients (iteration by iteration) between both periods (“after 1950” minus “before 1950”). Based on this new (delta) distribution, we evaluated a temporal change in the effect of each factor through the pMCMC value.

## Results

### Comparison between taxonomic groups and species habitats

**Fig 2:**
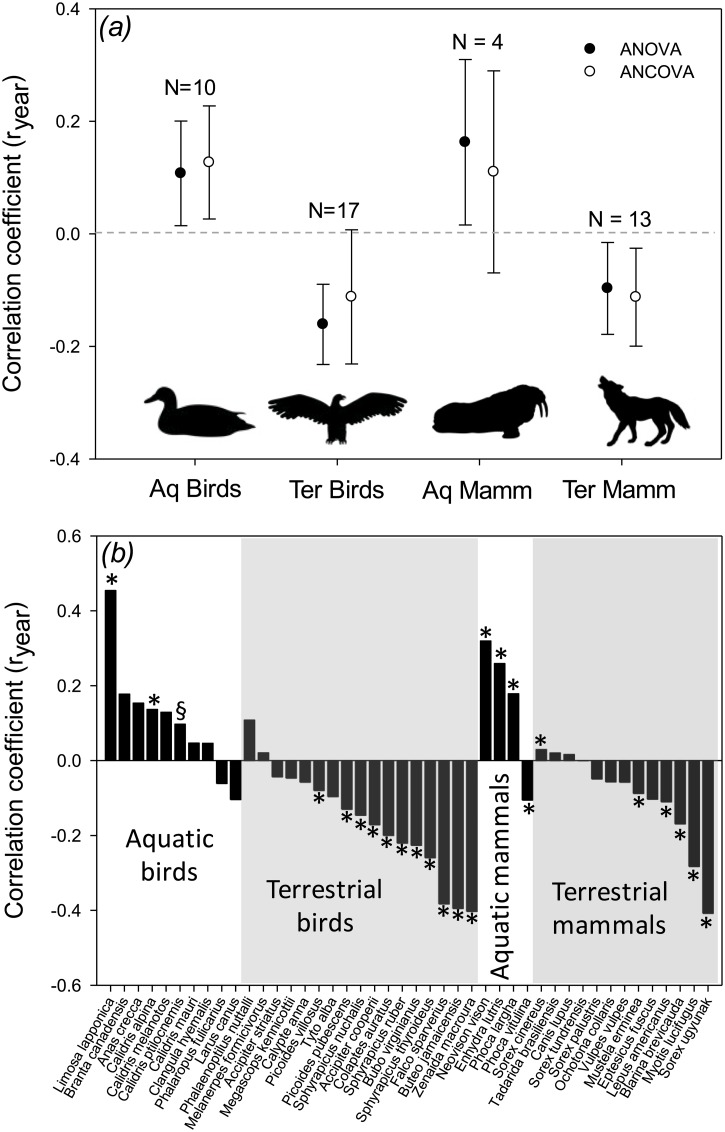
Correlation coefficient between body size and year of collection for *(a)* the four species groups that were compared, and *(b)* all the species included in the analysis. Aq Birds: aquatic birds, Ter Birds: terrestrial birds, AqMamm: aquatic mammals, Ter Mamm: terrestrial mammals. N = number of species in each group. Bars: 95% confidence intervals. * denotes P < 0.05 and § denotes P < 0.1.

Overall, six species increased their body mass, 21 species showed no significant changes in size, and 17 species decreased their body mass during the 20th century (Table A in S1 Appendix). The correlation between body mass and year of collection (r_year_) was similar for birds and mammals (ANOVA: F_1,40_ = 1.38, P = 0.25; ANCOVA: F_1,39_ = 0.01, P = 0.92; Fig 2a), but it strongly differs between aquatic and terrestrial species (ANOVA: F_1,40_ = 27.1, P < 0.001; ANCOVA: F_1,39_ = 14.8, P < 0.001; Fig 2a). Taken together, data for birds and mammals indicate that: (1) of 14 aquatic species, five showed a significant increase, eight showed a non-significant correlation, and one showed a significant decrease in body mass during the 20th century (Fig 2b); (2) of 30 terrestrial species, 16 showed a significant decrease, 13 showed a non-significant correlation, and one showed a significant increase in body mass during the 20th century (Fig 2b). That is, while most aquatic species increased or did not change body mass through the 20th century, most terrestrial species decreased in size. In line with these results, phylogenetic analyses indicate that, in addition to latitude and sex, there is a significant effect of the year of collection in all the models (Table 1). In addition, the model that included an interaction term between year of collection and habitat (model C) showed the best fit to the data (Table 1), and this interaction was significant, indicating again the contrasting pattern of variation between aquatic and terrestrial species.

**Table 1:** Posterior means (X), lower and upper confidence intervals (L95%CI and U95%CI, respectively), and Markov chain Monte Carlo p-value (pMCMC) obtained by phylogenetic generalized linear mixed models, with body mass as dependent variable, year of collection as the independent variable, species as a random factor, taxonomic group (class) and habitat as fixed factors, and (absolute) latitude, longitude, sex and length of the time series as covariates (model A). An interaction term between the year of collection and taxonomic group (class) or habitat was included in model B and C, respectively. For each model, the Deviance Information Criterion (DIC) score is provided in brackets; and for each factor, the level that was not dropped into the intercept is given in brackets.

	MODEL A (DIC = 58.051)				MODEL B (DIC = 58.053)				MODEL C (DIC = 57.986)			
	X	L95%CI	U95%CI	pMCMC	X	L95%CI	U95%CI	pMCMC	X	L95%CI	U95%CI	pMCMC
Intercept	4.6931	2.9896	6.3192	5.0E-04	5.0874	3.1025	7.1753	5.00E-04	-9.2593	-13.4541	-4.913	5.0E-04
Year	-0.0027	-0.0035	-0.0019	5.0E-04	-0.0029	-0.0039	-0.0020	5.00E-04	0.0042	0.0021	0.0063	5.0E-04
Class (Mammals)	-0.0782	-0.4126	0.2064	0.622	-1.2422	-4.9069	2.3277	0.514	-0.0104	-0.3210	0.2813	0.947
Habitat (Terrestrial)	0.1188	-0.0755	0.3193	0.252	0.1141	-0.0753	0.3165	0.259	16.7406	12.0595	21.3008	5.0E-04
Latitude	0.0113	0.0091	0.0136	5.0E-04	0.0114	0.0090	0.0136	5.00E-04	0.0112	0.0088	0.0134	5.0E-04
Longitude	7.2E-06	-0.0003	0.0003	0.949	2.1E-05	-0.0004	0.0003	0.900	-0.0002	-0.0005	0.0002	0.327
Sex (male)	0.0462	0.0186	0.0719	0.002	0.0453	0.0172	0.0710	5.00E-04	0.0439	0.0164	0.0698	5.0E-04
Time series length	0.0007	-0.0052	0.0063	0.806	0.0007	-0.0048	0.0064	0.822	0.0030	-0.0028	0.0086	0.307
Interaction term	-----	-----	-----	-----	0.0006	-0.0011	0.0025	0.529	-0.0084	-0.0108	-0.0061	5.0E-04

### Temporal variation in the temporal response

**Fig 3:**
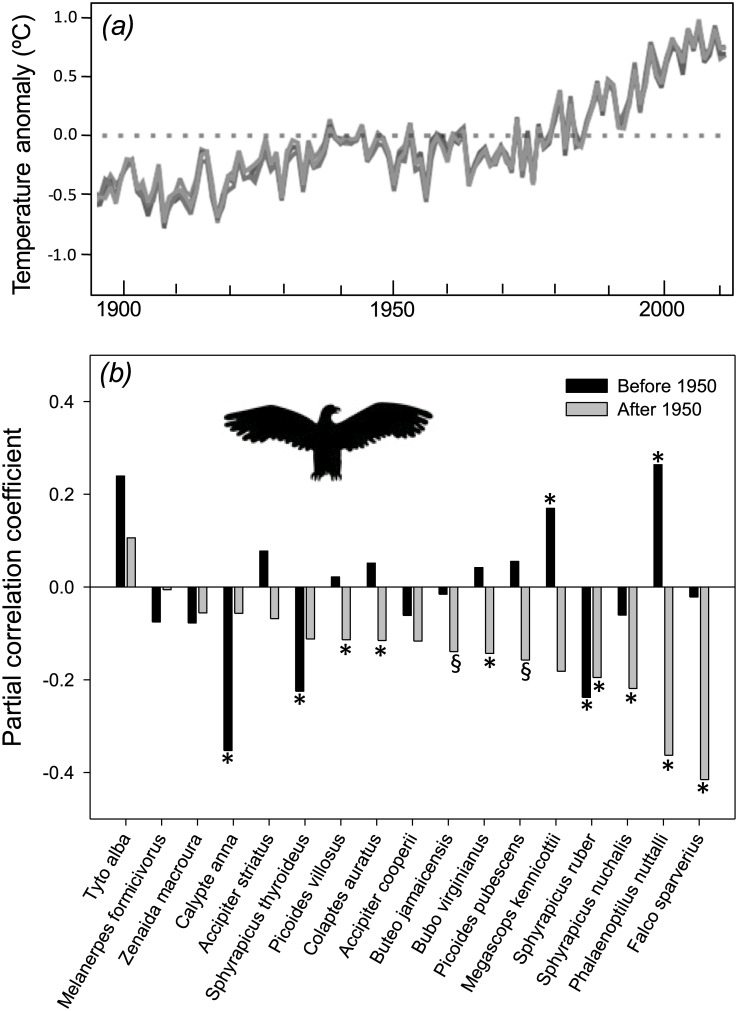
*(a)* Change in land surface temperature for the last century (modified from [29]), and *(b)* correlation coefficient between body size and year of collection for terrestrial bird species during the first (before 1950) and second (after 1950) half of the 20^th^ century. * denotes P < 0.05 and § denotes P < 0.1.

As mentioned above, the rate of change in global temperature was greater for the second half of the 20^th^ century than for the first half (Fig 3a). In line with this, we found that before 1950 two terrestrial bird species showed a significant increase in body mass, twelve species showed no significant changes in size, and three species showed a significant decrease in body mass (Fig 3b; Table B in S1 Appendix). However, after 1950 we found that eight species showed a significant decrease in body mass, while the remaining nine species showed no significant correlation between body mass and year of collection (Fig 3b; Table B in S1 Appendix). Accordingly, the mean value of r_year_ differed between both periods of time (t_16_ = 2.46, P = 0.03), being very close to zero for the first half of the 20^th^century (X = -0.01, SD = 0.16) and negative for the second half of this century (X = -0.14, SD = 0.12). In the same vein, phylogenetic analyses indicate that regression coefficient for the year of collection did not differ from zero for the first half of the 20^th^ century, but was negative and highly significant for the second half of that century (Table 2). Moreover, the distribution of the difference of the regression coefficients for the year of collection between both periods of time (“after 1950” minus “before 1950”) was negative and statistically significant (Table 2).

**Table 2:** Posterior means (X), lower and upper confidence intervals (L95%CI and U95%CI, respectively), and Markov chain Monte Carlo p-value (pMCMC) obtained by phylogenetic generalized linear mixed models including data before 1950, after 1950, and comparing both periods of time. For the factor “sex”, the level that was not dropped into the intercept is provided in brackets.

	Before 1950				After 1950					Δ Periods		
	X	L95%CI	U95%CI	pMCMC	X	L95%CI	U95%CI	pMCMC	X	L95%CI	U95%CI	pMCMC
Intercept	-2.9162	-11.4821	5.4466	0.503	11.2687	6.4670	15.9670	0.001	14.1849	5.3003	24.9950	0.008
Year	0.0007	-0.0034	0.0049	0.711	-0.0063	-0.0084	-0.0041	5.0E-04	-0.0070	-0.0121	-0.0025	0.003
Latitude	0.0528	0.0463	0.0592	5.0E-04	0.0402	0.0341	0.0464	5.0E-04	-0.0126	-0.0214	-0.0036	0.007
Longitude	0.0028	-0.0022	0.0079	0.277	0.0017	-0.0007	0.0042	0.185	-0.0011	-0.0069	0.0044	0.724
Sex (male)	-0.1321	-0.2111	-0.0574	0.001	-0.0796	-0.1522	-0.0046	0.037	0.0525	-0.0577	0.1571	0.353

## Discussion

Importantly, nearly all the data considered in our analyses are from specimens from the middle and high latitudes along the west coast and western North American sub-continent (Figure D in S1 Appendix). Although this fact may constrain generalization of our results, ambient temperature in this area has increased between 1°C and 2°C through the 20^th^century [29]. Thus, the changes in body size that were observed here could be linked, at least in theory, to direct and indirect effects of changes in ambient temperature. Three major results emerge from the present study. First, only subtle (non significant) differences in the temporal changes in body mass between birds and mammals were observed. Second, temporal changes in body mass recorded for aquatic species were different from those recorded for terrestrial species. Third, the temporal reduction in body size observed in terrestrial birds was greater for the second half of the 20^th^ century than for the first half.

The first result is interesting because previously published data suggest that significant reductions in body size appear to be more common in birds, while significant increases in size appear to be more common in mammals [5, 13]. However, in contrast to our study in which aquatic species represent 32% of species, previous works were conducted almost exclusively on terrestrial species (> 95%). Thus, it could be possible that the discrepancy between our study and previous works is related to a difference in the relative number of species that occur in aquatic habitats. In this sense, a close inspection of our results for terrestrial species indicates that the proportion of species that showed a significant reduction in body size is higher in birds (64.7%) than in mammals (38.5%). We think that this taxonomic difference in terrestrial habitat could be related to differences in biology and especially linked to the energetic argument. That is, because birds have higher metabolic rates for a given body mass than mammals [30], the selective pressure for increasing heat dissipation abilities (i.e., smaller sizes) could be higher in the former group.

The second result, in our opinion, is the most interesting outcome of the present study. In short, we found that while most of the aquatic species increased or did not change in body mass through the 20^th^ century, most terrestrial species decreased in size. We believe that this is not the consequence of a single physiological or ecological factor acting differentially between the two habitats, but the result of an imbalance in the number of factors promoting increase and decrease in size between aquatic and terrestrial systems. In addition, given that species classified as aquatic in the present study range from semi-aquatic to fully aquatic, the relevance of each factor enlisted below could change to a greater extent according to the biology of individual species. First, the fact that water thermal conductivity is about 23 times greater than air thermal conductivity means that thermal stability is much greater in aquatic than in terrestrial systems [31]. Consequently, extreme events during which ambient temperature rises above the upper limit of the thermoneutral zone –which usually is between 30°C and 40°C [32]–could be fairly common on land but rare in aquatic habitats (and surely nonexistent in aquatic habitats at higher latitudes). Along these lines, a decrease in body size due to the energetic argument depicted by Bergmann [8] is not expected for most aquatic species. Interestingly, an increase in body size related to a rise in the number of extremely hot days, due to a positive relationship between size and water conservation ability, also has been proposed for terrestrial birds, but only for some species inhabiting arid environments [33–34]. Second, the greater thermal conductivity of water, when combined with the fact that mean ambient temperatures usually are below the lower limit of the thermoneutral zone [32], implies that the costs of maintenance usually are higher in aquatic endotherms than in terrestrial ones [30]. Then, even a slight rise in water temperature could represent an important savings in the amount of energy needed for thermoregulation, and thus, an increase in the amount of energy that aquatic species could devote to body growth [35]. Third, given that growth rate in primary producers is positively related to ambient temperature, global warming is expected to result in an increase in net primary productivity (NPP, a rough proxy of overall food availability in a system). However, the real effect of temperature on NPP usually changes between geographic areas, depending on water availability in terrestrial systems [17] and on nutrient availability (e.g., nitrogen and phosphorous) in aquatic systems [36–37]. In this sense, while terrestrial species in our data set belong to areas that strongly differ in water availability (from desert to boreal forest), practically all the aquatic species occur in high latitude coastal and inland systems, where a positive effect of temperature on NPP is expected [37]. Thus, at least in our study, temporal changes in food availability probably differed among terrestrial species, but consistently increased for aquatic species. Fourth, the reduction in the mean body size recently recorded for several fish assemblages, due to size-at-age, population age-structure, and species compositional shifts [1,3, 38], may have led to an increase in the number of prey types consumed by aquatic birds and mammals. In addition, the reduction in the abundance of larger individuals of some large species of fish, mostly caused by human exploitation (e.g., [39]), may also reduce the selective pressure of competition acting on some aquatic endotherms (e.g., large predators). Finally, habitat fragmentation heavily impacts terrestrial systems and is expected to negatively affect body size, because home range directly affects the amount of resources available to each species [40]. Thus, we believe that differences in the factors favoring increases and decreases in body size between aquatic and terrestrial habitats could be responsible for the dissimilar pattern of body size change. However, research aimed at testing this idea is necessary, especially because relatively few aquatic species have been analyzed.

Finally, we found that temporal decrease in body size for terrestrial birds was noticeably greater during the second half than during the first half of the 20^th^ century. In effect, both conventional and phylogenetic analyses indicate that mean slope value between body mass and year of collection was very close to zero for the first half of 20^th^ century, but negative for the second half of that century. Although seldom explored, temporal variation in body size changes should be expected because global warming, as well as other anthropogenic modifications of habitats (fragmentation, pollution, etc), increased noticeably after 1950. Thus, our results reinforce the idea that, at least for some groups of animals, human activities are behind the recorded changes in body size patterns (see [41]). Nevertheless, further investigations are needed to understand how human-caused phenotypic changes are linked to population process, and hence, to know if they really comprise a useful piece of information when assessing “health status” at the population or species levels (see [42]). The vast digital resource on trait variability now available through on-line museum databases provides opportunities to explore the effects of environmental change on diverse organisms [43].

## Supporting information

S1 Appendix Appendix with supplementary Tables A and B, and supplementary Figures A, B, C and D. (DOCX)

S1 Dataset Compilation of data on body size change from 46 published studies, comprising a total of 369 populations of birds and mammals. Studies reporting the changes due to experimental manipulations, colonization of islands, or spanning over < 10 years were excluded.
(XLSX)

S2 Dataset Body mass, year of collection, latitude, longitude and sex for each individual analyzed in the present study (20,496 records). (XLSX)
