# Supplementary material for: Climate change and body size trends in aquatic and terrestrial endotherms: Does habitat matter?
Source: PLoS One. 2017 Aug 16;12(8):e0183051. doi: 10.1371/journal.pone.0183051 (PMC5558942; doi:10.1371/journal.pone.0183051)

**Appendix**

**Table A.** List of the species included in our analysis indicating sample size (n), mean body mass (and its standard error) in our sample (m_b_), partial correlation coefficient for year of collection (r_year_), latitude (r_lat_), longitude (r_long_) and sex (r_sex_), year of the earliest and latest record (Y_i_ and Y_f_, respectively), and the lowest and highest latitude of collection (L_l_ and L_h_, respectively). * denotes P < 0.05 and § denotes P < 0.1.

| **Species** | **Class** | **Habitat** | **N** | **m_b_ (g)** | **r_year_** | **r_lat_** | **rl_ong_** | **r_sex_** | **Y_i_** | **Y_f_** | **L_l_** | **L_h_** |
| --- | --- | --- | --- | --- | --- | --- | --- | --- | --- | --- | --- | --- |
| *Accipiter cooperii* | Aves | Terrestrial | 146 | 361.2 (8.7) | -0.172* | 0.115 | 0.220* | 0.530* | 1915 | 2014 | 20.8 | 49.4 |
| *Accipiter striatus* | Aves | Terrestrial | 205 | 137.0 (2.9) | -0.044 | 0.116 | 0.083 | 0.773* | 1914 | 2014 | 18.3 | 64.9 |
| *Anas crecca* | Aves | Aquatic | 127 | 349.5 (4.8) | 0.153* | 0.201 | -0.077 | -0.227* | 1917 | 2012 | 23.2 | 70.8 |
| *Blarina brevicauda* | Mammalia | Terrestrial | 352 | 17.9 (0.2) | -0.169* | 0.233* | -0.209* | 0.311* | 1933 | 2013 | 35.3 | 48.2 |
| *Branta canadensis* | Aves | Aquatic | 107 | 2404.9 (127.2) | 0.178 | -0.284* | -0.148 | -0.133 | 1919 | 2013 | 36.6 | 68.9 |
| *Bubo virginianus* | Aves | Terrestrial | 292 | 1135.3 (17.4) | -0.227* | 0.618* | 0.365* | 0.310* | 1915 | 2014 | 23.1 | 64.9 |
| *Buteo jamaicensis* | Aves | Terrestrial | 222 | 982.7 (16.7) | -0.395* | -0.023 | 0.147* | 0.525* | 1915 | 2014 | 19.3 | 71.3 |
| *Calidris alpina* | Aves | Aquatic | 377 | 54.1 (0.5) | 0.137* | -0.034 | -0.127* | 0.219* | 1918 | 2010 | 23.4 | 71.4 |
| *Calidris mauri* | Aves | Aquatic | 173 | 25.5 (0.3) | 0.047 | 0.009 | -0.126 | 0.278* | 1918 | 2010 | -13.8 | 71.3 |
| *Calidris melanotos* | Aves | Aquatic | 138 | 68.4 (1.9) | 0.129 | 0.131 | -0.006 | -0.415* | 1919 | 2013 | -37.3 | 73.5 |
| *Calidris ptilocnemis* | Aves | Aquatic | 323 | 72.8 (0.6) | 0.097§ | -0.054 | -0.191* | 0.393* | 1955 | 2013 | 51.4 | 65.8 |
| *Calypte anna* | Aves | Terrestrial | 130 | 4.1 (0.05) | -0.058 | -0.101 | -0.026 | -0.436* | 1914 | 2010 | 30.7 | 40.3 |
| *Canis lupus* | Mammalia | Terrestrial | 609 | 36945.9 (332.4) | 0.016 | 0.210* | 0.159* | 0.342* | 1950 | 2013 | 32.2 | 69.4 |
| *Clangula hyemalis* | Aves | Aquatic | 117 | 716.4 (10.9) | 0.046 | 0.235* | -0.010 | -0.499* | 1924 | 2011 | 33.2 | 71.4 |
| *Colaptes auratus* | Aves | Terrestrial | 931 | 135.2 (0.5) | -0.200* | 0.121* | -0.123* | -0.082* | 1914 | 2014 | 16.1 | 64.9 |
| *Enhydra lutris* | Mammalia | Aquatic | 683 | 20607.7 (327.2) | 0.259* | -0.353* | -0.068 | 0.232* | 1967 | 2009 | 51.5 | 61.1 |
| *Eptesicus fuscus* | Mammalia | Terrestrial | 392 | 106.0 (0.5) | -0.103 | -0.080 | 0.078 | -0.257* | 1913 | 2014 | 32.6 | 45.4 |
| *Falco sparverius* | Aves | Terrestrial | 279 | 100.2 (1.2) | -0.383* | 0.316* | 0.179* | 0.239* | 1915 | 2013 | 2.3 | 64.9 |
| *Larus canus* | Aves | Aquatic | 108 | 374.0 (6.4) | -0.104 | 0.035 | 0.408* | -0.299* | 1919 | 2009 | 36.6 | 71.3 |
| *Lepus americanus* | Mammalia | Terrestrial | 557 | 1373.0 (15.3) | -0.110* | 0.028 | -0.143* | -0.239* | 1950 | 2013 | 43.0 | 69.4 |
| *Limosa lapponica* | Aves | Aquatic | 106 | 263.4 (4.8) | 0.454* | 0.059 | -0.277* | 0.553* | 1951 | 2011 | 35.1 | 71.4 |
| *Megascops kennicottii* | Aves | Terrestrial | 293 | 137.2 (1.6) | -0.047 | 0.477* | -0.101 | 0.405* | 1916 | 2009 | 23.4 | 58.3 |
| *Melanerpes formicivorus* | Aves | Terrestrial | 261 | 72.9 (0.6) | 0.021 | 0.268 | -0.049 | -0.170* | 1914 | 2011 | 2.1 | 41.2 |
| *Mustela erminea* | Mammalia | Terrestrial | 359 | 104.0 (2.4) | -0.088* | 0.470* | 0.040 | 0.481 | 1950 | 2014 | 35.8 | 71.3 |
| *Myotis lucifugus* | Mammalia | Terrestrial | 318 | 82.7 (0.6) | -0.283* | 0.249 | -0.103 | -0.182* | 1936 | 2010 | 34.3 | 65.8 |
| *Neovison vison* | Mammalia | Aquatic | 385 | 102.7 (16.9) | 0.319* | 0.130* | -0.132* | 0.530* | 1950 | 2014 | 29.7 | 66.6 |
| *Ochotona collaris* | Mammalia | Terrestrial | 227 | 123.6 (1.7) | -0.057 | -0.100 | -0.065 | 0.040 | 1951 | 2013 | 59.6 | 65.5 |
| *Phalaenoptilus nuttalli* | Aves | Terrestrial | 235 | 44.7 (0.5) | 0.108 | 0.213* | 0.005 | 0.020 | 1915 | 2012 | 23.4 | 45.3 |
| *Phalaropus fulicarius* | Aves | Aquatic | 281 | 49.9 (0.6) | -0.061 | 0.374* | 0.099 | 0.322* | 1918 | 2009 | 23.4 | 71.4 |
| *Phoca larga* | Mammalia | Aquatic | 276 | 51665.2 (1778.6) | 0.179* | -0.247* | 0.034 | 0.185* | 1956 | 2012 | 51.5 | 70.6 |
| *Phoca vitulina* | Mammalia | Aquatic | 761 | 56650.2 (906.6) | -0.106* | -0.127* | 0.172* | 0.100* | 1949 | 2009 | 37.8 | 61.5 |
| *Picoides pubescens* | Aves | Terrestrial | 242 | 26.6 (0.2) | -0.130* | 0.321* | -0.077 | -0.101 | 1915 | 2014 | 29.6 | 64.9 |
| *Picoides villosus* | Aves | Terrestrial | 494 | 66.1 (0.5) | -0.080* | 0.826* | 0.010 | -0.344* | 1914 | 2013 | 9.8 | 64.9 |
| *Sorex cinereus* | Mammalia | Terrestrial | 7689 | 3.9 (0.01) | 0.029* | -0.079* | 0.034* | -0.011 | 1930 | 2014 | 35.8 | 68.6 |
| *Sorex palustris* | Mammalia | Terrestrial | 120 | 10.5 (0.2) | -0.049 | 0.081 | 0.261 | 0.168 | 1951 | 2012 | 34.0 | 65.2 |
| *Sorex tundrensis* | Mammalia | Terrestrial | 541 | 6.7 (0.07) | 0.000 | 0.029 | -0.177* | 0.031 | 1950 | 2013 | 57.2 | 70.3 |
| *Sorex ugyunak* | Mammalia | Terrestrial | 135 | 3.9 (0.08) | -0.408* | -0.047 | -0.025 | 0.084 | 1951 | 2010 | 65.4 | 71.4 |
| *Sphyrapicus nuchalis* | Aves | Terrestrial | 282 | 45.9 (0.3) | -0.146* | -0.003 | -0.046 | -0.059 | 1917 | 2013 | 29.0 | 52.8 |
| *Sphyrapicus ruber* | Aves | Terrestrial | 396 | 48.2 (0.2) | -0.221* | 0.189* | -0.163 | -0.117* | 1914 | 2012 | 31.0 | 59.2 |
| *Sphyrapicus thyroideus* | Aves | Terrestrial | 179 | 50.1 (0.4) | -0.260* | -0.074 | -0.202* | -0.033 | 1915 | 2010 | 29.5 | 48.4 |
| *Tadarida brasiliensis* | Mammalia | Terrestrial | 154 | 94.2 (0.4) | 0.020 | -0.238* | 0.208* | -0.025 | 1939 | 2014 | 29.4 | 40.9 |
| *Tyto alba* | Aves | Terrestrial | 110 | 419.5 (7.4) | -0.096 | -0.176 | -0.170* | 0.234 | 1918 | 2014 | -34.9 | 51.6 |
| *Vulpes vulpes* | Mammalia | Terrestrial | 265 | 3895.8 (53.2) | -0.058 | 0.509* | 0.463* | 0.385* | 1941 | 2013 | 34.2 | 70.2 |
| *Zenaida macroura* | Aves | Terrestrial | 119 | 110.5 (2.0) | -0.403* | 0.382* | 0.485* | -0.202* | 1917 | 2013 | 16.3 | 56.6 |

**Table B. S**ample size (n), partial correlation coefficient for year of collection (r_year_), latitude (r_lat_), longitude (r_long_) and sex (r_sex_), year of the earliest and latest record (Y_i_ and Y_f_, respectively), and lowest and highest latitude of collection (L_l_ and L_h_, respectively) for terrestrial bird species, at two different period of time (before and after 1950). * denotes P < 0.05 and § denotes P < 0.1.

| **Species** | **Before 1950** | | | | | | | | **After 1950** | | | | | | | | |
| --- | --- | --- | --- | --- | --- | --- | --- | --- | --- | --- | --- | --- | --- | --- | --- | --- | --- |
|  | **n** | **r_year_** | **r_lat_** | **r_long_** | **r_sex_** | **Y_i_** | **L_l_** | **L_h_** | **n** | **r_year_** | **r_lat_** | **r_long_** | **r_sex_** | **Y_f_** | **L_l_** | **L_h_** |  |
| *A. cooperii* | 44 | -0.061 | 0.342* | 0.280* | 0.500* | 1915 | 20.8 | 49.4 | 102 | -0.117 | -0.006 | 0.265* | 0.538* | 2014 | 28.6 | 47.0 |  |
| *A. striatus* | 73 | 0.078 | 0.108 | 0.031 | 0.877* | 1914 | 18.3 | 59.6 | 132 | -0.068 | 0.165* | 0.117 | 0.708* | 2014 | 29.0 | 64.9 |  |
| *B. virginianus* | 122 | 0.042 | 0.566* | 0.353* | 0.502* | 1915 | 23.1 | 59.6 | 170 | -0.143* | 0.640* | 0.398* | 0.198* | 2014 | 28.7 | 64.9 |  |
| *B. jamaicensis* | 90 | -0.016 | 0.040 | 0.107 | 0.624* | 1915 | 19.3 | 59.6 | 132 | -0.139§ | -0.076 | 0.153 | 0.535* | 2014 | 29.0 | 71.3 |  |
| *C. anna* | 47 | -0.353* | 0.001 | 0.045 | -0.381* | 1914 | 30.7 | 40.3 | 83 | -0.057 | -0.487§ | -0.331 | -0.441* | 2010 | 32.7 | 39.0 |  |
| *C. auratus* | 284 | 0.052 | 0.163* | -0.178* | -0.063 | 1914 | 16.1 | 59.6 | 647 | -0.115* | 0.116* | -0.044 | -0.101* | 2014 | 19.0 | 64.9 |  |
| *F. sparverius* | 146 | -0.021 | 0.590* | 0.386* | 0.258* | 1915 | 3.2 | 55.3 | 133 | -0.415* | 0.204* | 0.041 | 0.210* | 2013 | 2.3 | 64.9 |  |
| *M. kennicottii* | 203 | 0.170* | 0.329* | -0.262* | 0.443* | 1916 | 23.4 | 58.3 | 90 | -0.182* | 0.378§ | -0.104 | 0.459* | 2009 | 25.8 | 41.4 |  |
| *M. formicivorus* | 132 | -0.075 | 1.371* | 0.843* | -0.122 | 1914 | 2.1 | 41.2 | 129 | -0.006 | -1.467* | -1.616* | -0.186* | 2011 | 2.2 | 39.2 |  |
| *P. nuttalli* | 180 | 0.264* | 0.186* | -0.063 | 0.010 | 1915 | 23.4 | 45.3 | 55 | -0.363* | 0.229 | 0.095 | 0.084 | 2012 | 26.1 | 43.5 |  |
| *P. pubescens* | 115 | 0.055 | 0.260* | -0.067 | -0.092 | 1915 | 35.4 | 59.6 | 127 | -0.157§ | 0.342* | -0.108 | -0.106 | 2014 | 29.6 | 64.9 |  |
| *P. villosus* | 345 | 0.022 | 0.777* | 0.027 | -0.379* | 1914 | 16.1 | 59.6 | 149 | -0.114* | 0.852* | -0.067 | -0.286* | 2013 | 9.8 | 64.9 |  |
| *S. nuchalis* | 92 | -0.060 | 0.034 | -0.119 | 0.187 | 1917 | 31.0 | 52.8 | 190 | -0.219* | -0.019 | -0.016 | -0.199* | 2013 | 29.0 | 48.9 |  |
| *S. ruber* | 166 | -0.238* | 0.126 | -0.278* | -0.072 | 1914 | 31.0 | 57.6 | 230 | -0.195* | 0.378* | 0.003 | -0.175* | 2012 | 35.6 | 59.2 |  |
| *S. thyroideus* | 90 | -0.225* | -0.157 | -0.168 | -0.066 | 1915 | 30.9 | 45.0 | 89 | -0.112 | 0.072 | -0.144 | -0.035 | 2010 | 29.5 | 48.4 |  |
| *T. alba* | 24 | 0.239 | 0.176 | -0.060 | 0.331 | 1918 | 31.0 | 42.4 | 86 | 0.106 | -0.206 | -0.126 | 0.235* | 2014 | -34.9 | 51.6 |  |
| *Z. macroura* | 65 | -0.077 | 0.370§ | 0.178 | -0.169 | 1917 | 16.3 | 56.6 | 54 | -0.055 | 0.201 | 0.543 | -0.206 | 2013 | 28.7 | 48.4 |  |

**Fig A.** Relationship between body size and year of collection for the six species that were not included in the analysis due to the uneven distribution of data across time.


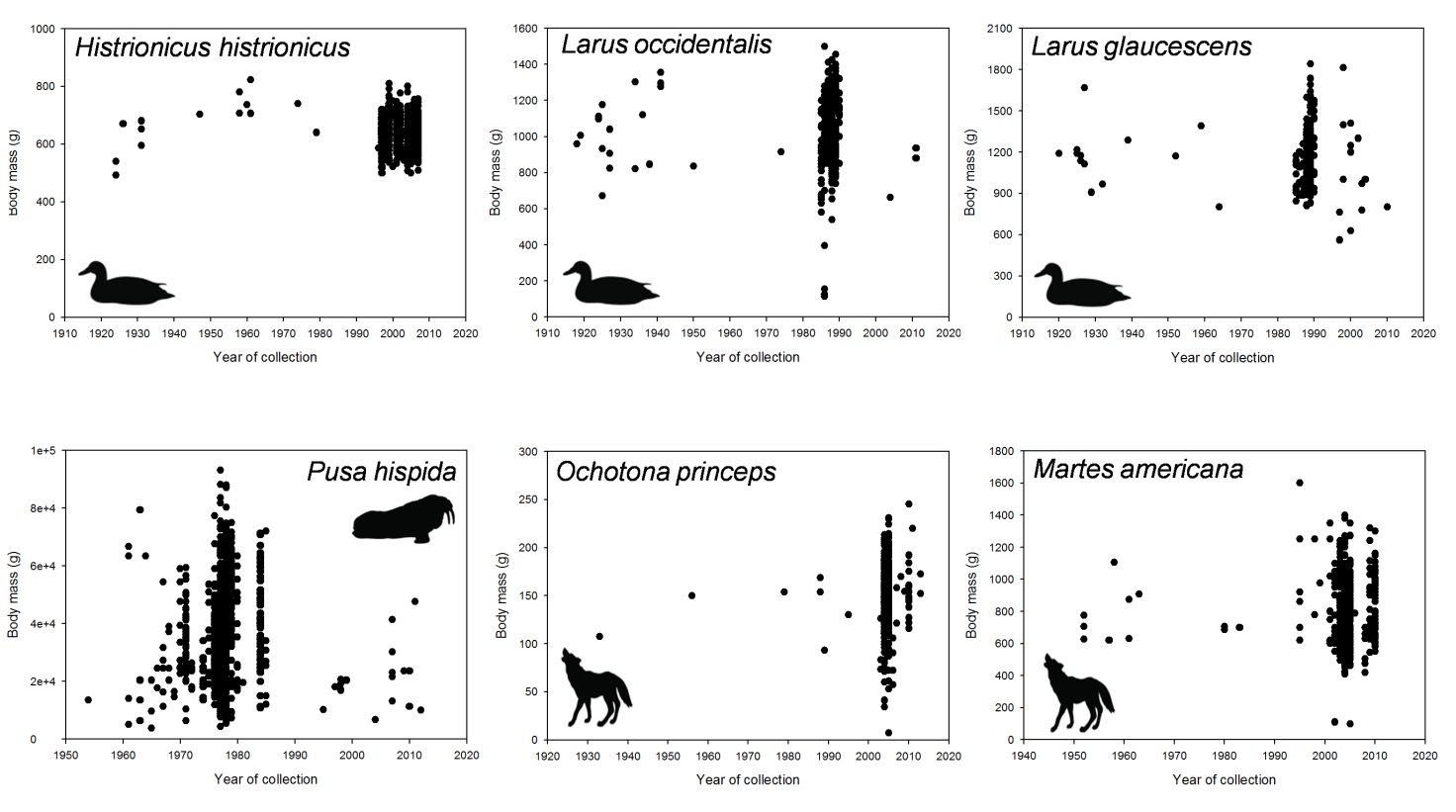


**Fig B.** Relationship between body size and year of collection, together with a world map showing the sites of collection, for each species included in the analysis.

**
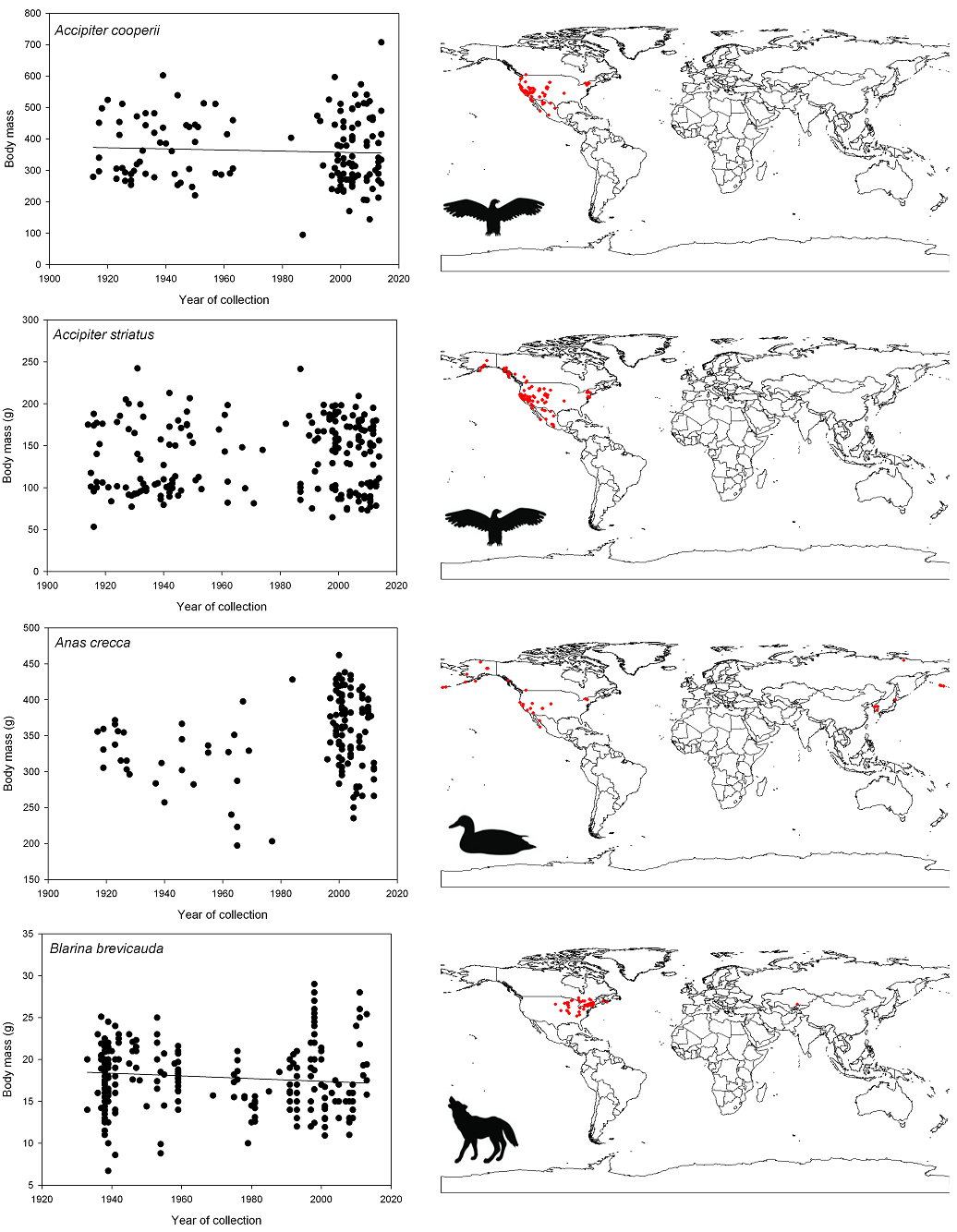
**

**Fig B (cont).**

**
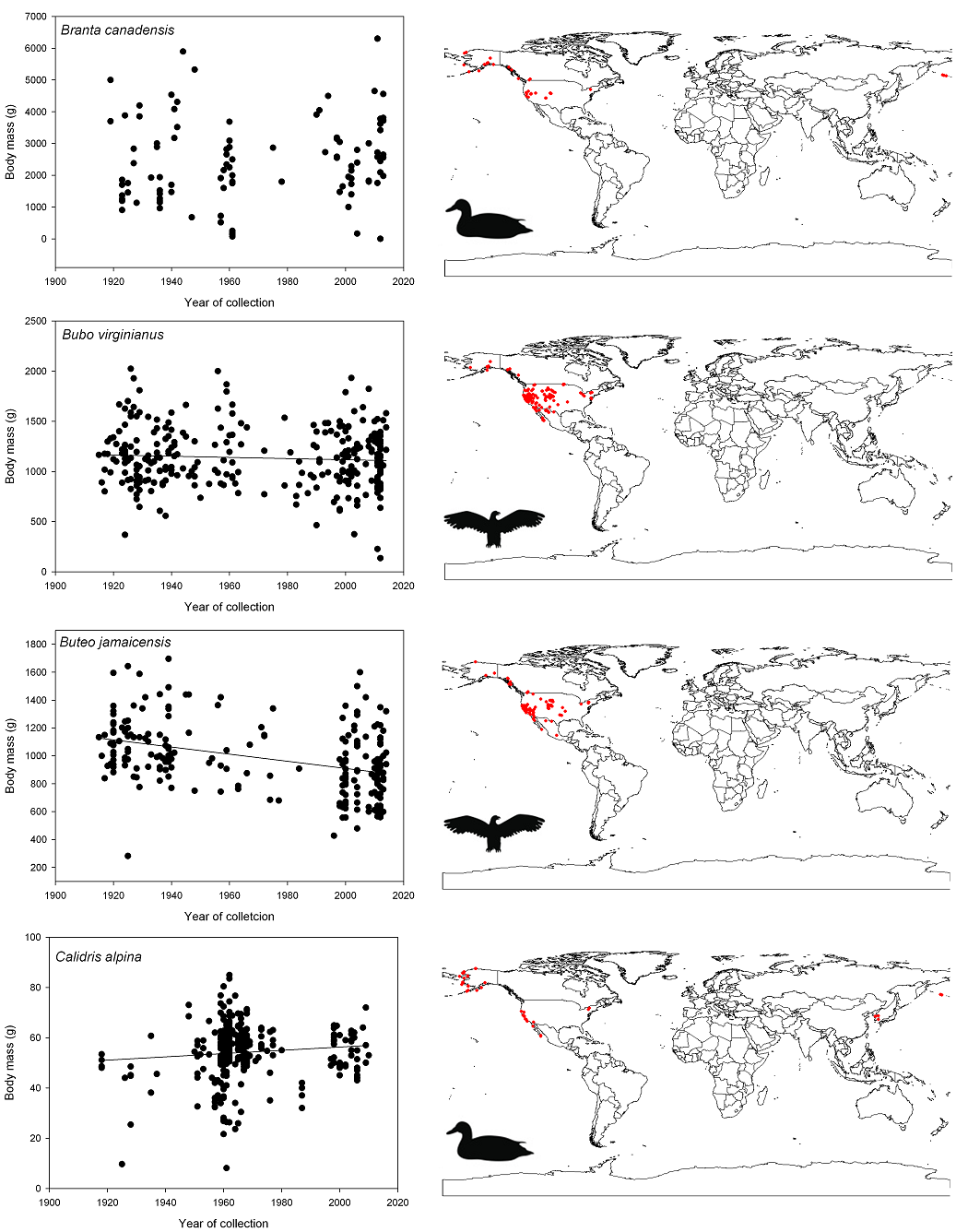
**

**Fig B (cont).**

**
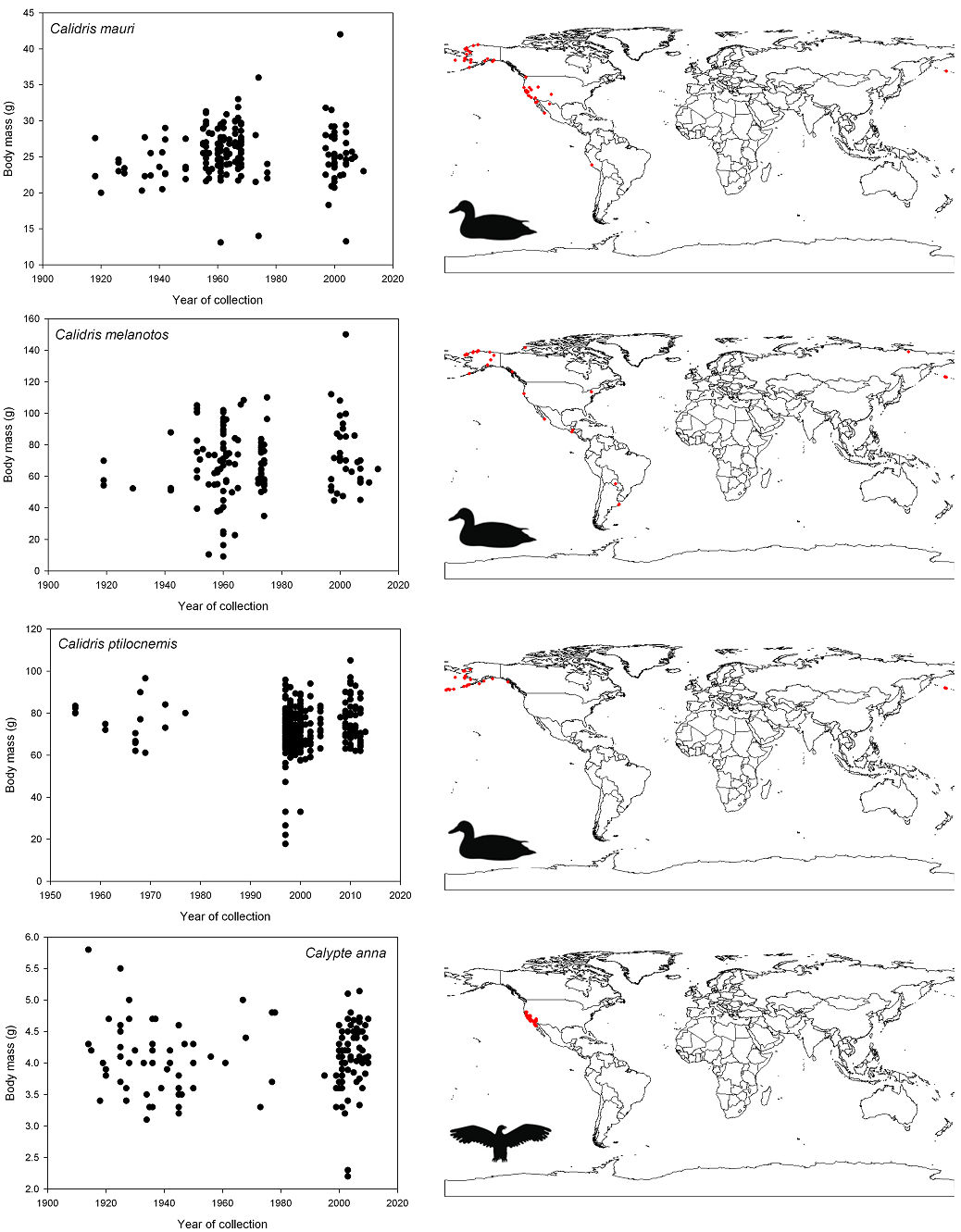
**

**Fig B (cont).**

**
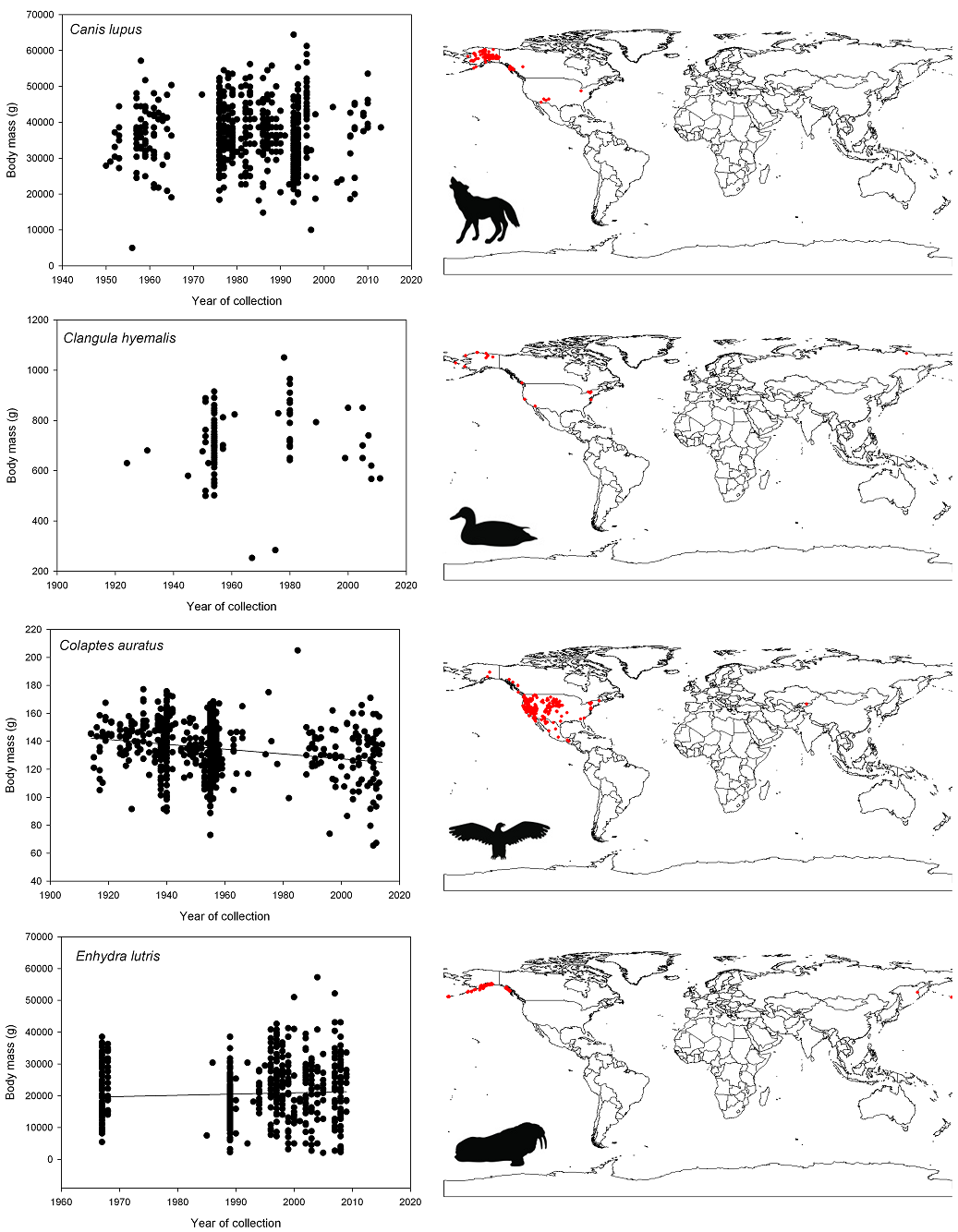
**

**Fig B (cont).**

**
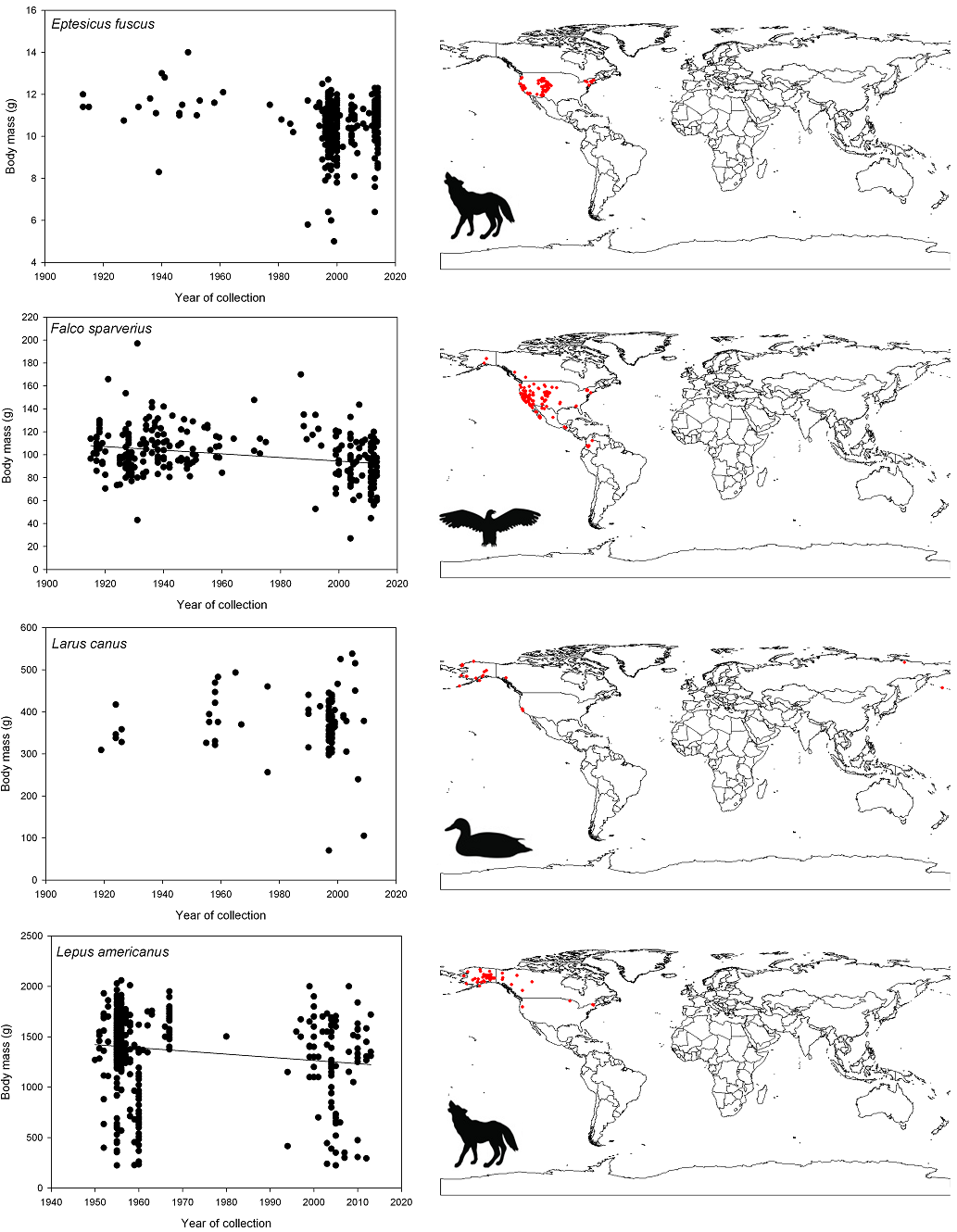
**

**Fig B (cont).**

**
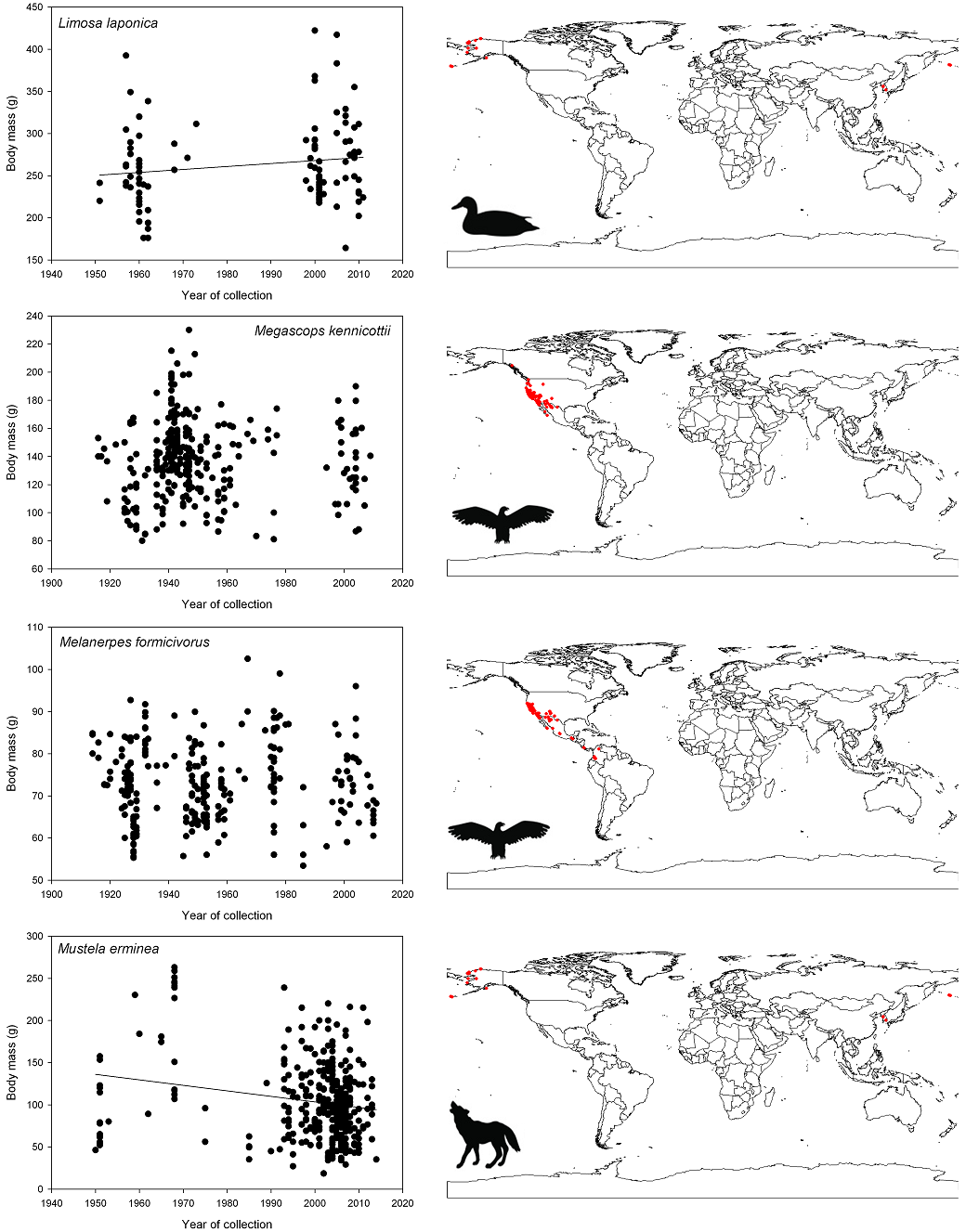
**

**Fig B (cont).**

**
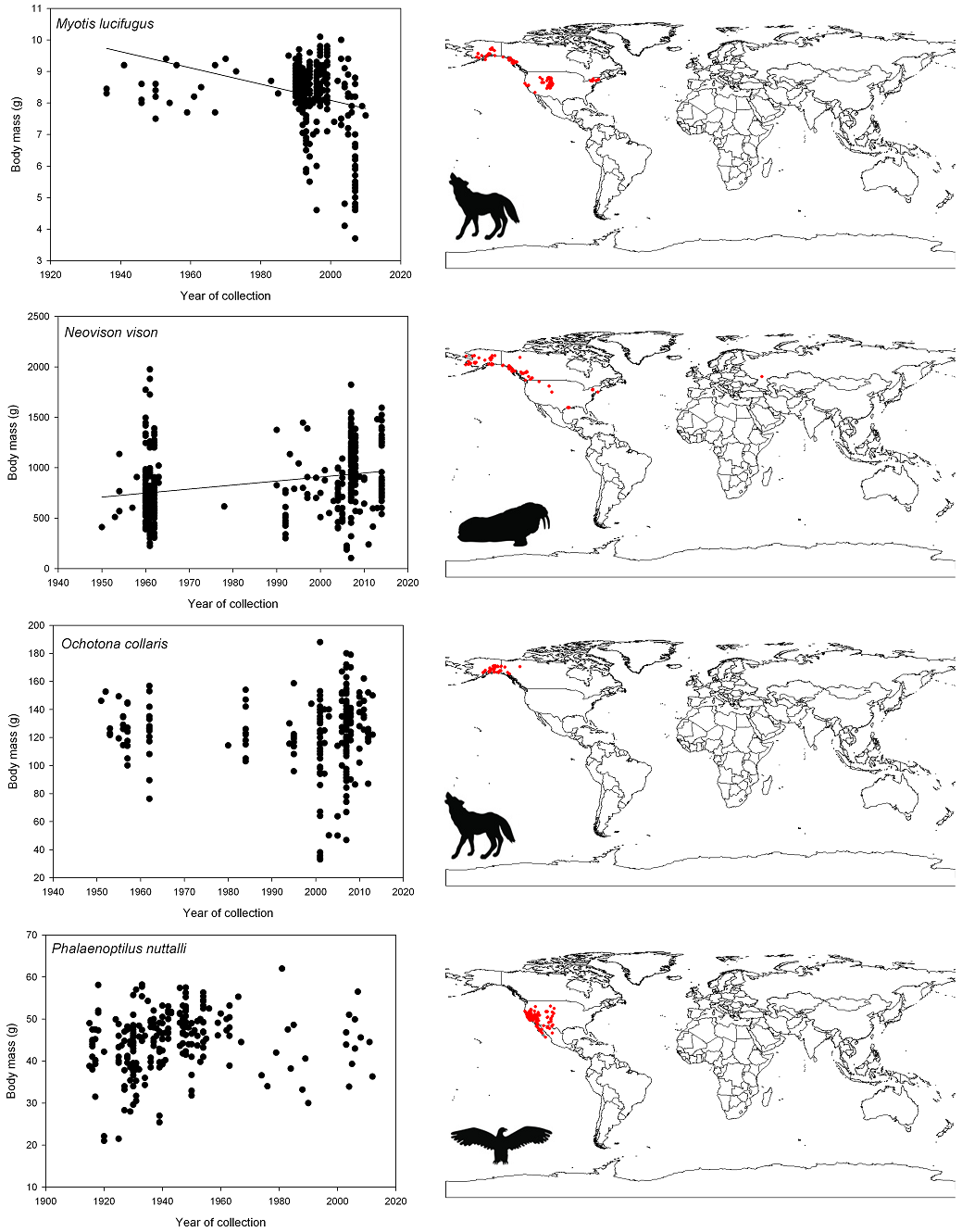
**

**Fig B (cont).**

**
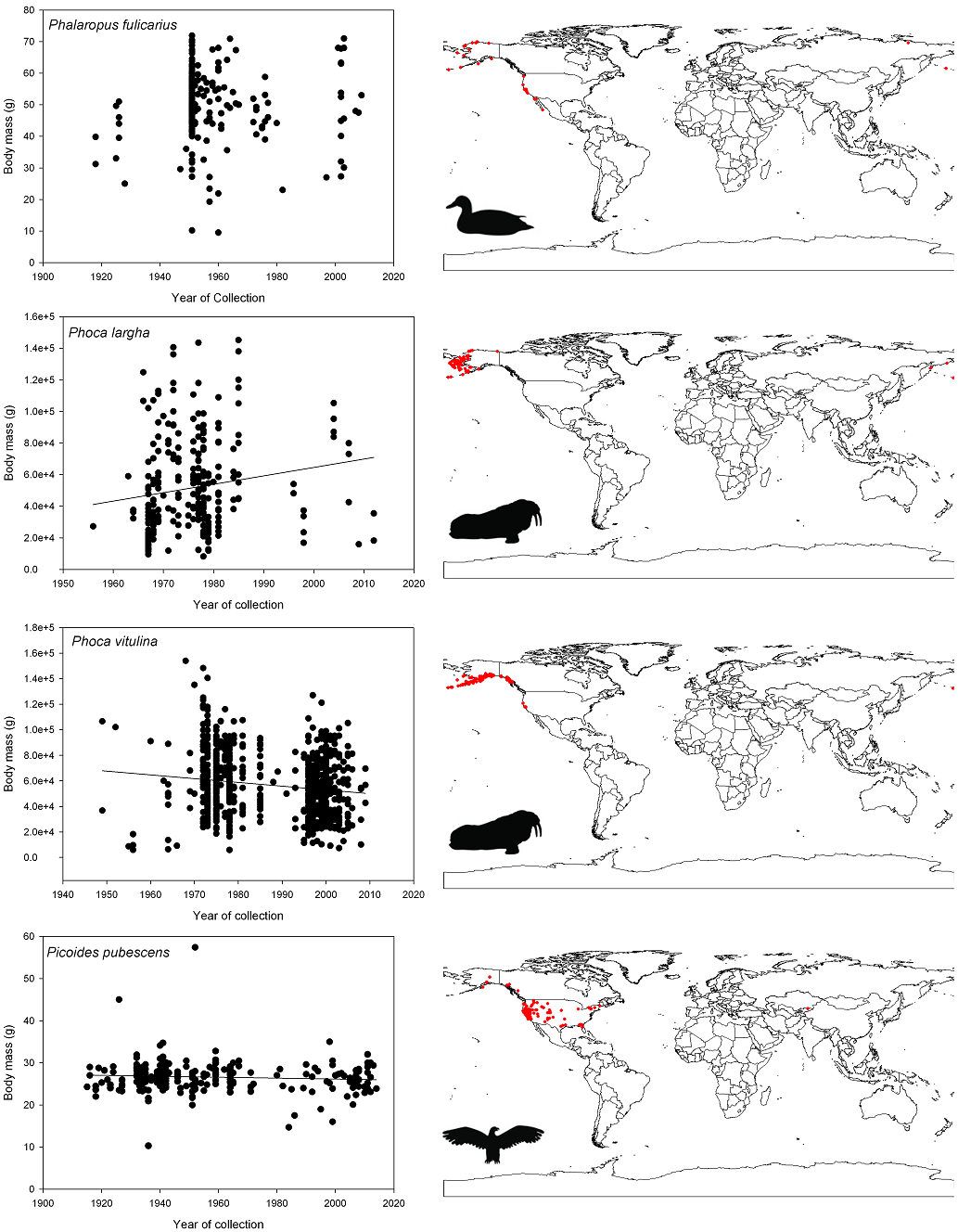
**

**Fig B (cont).**

**
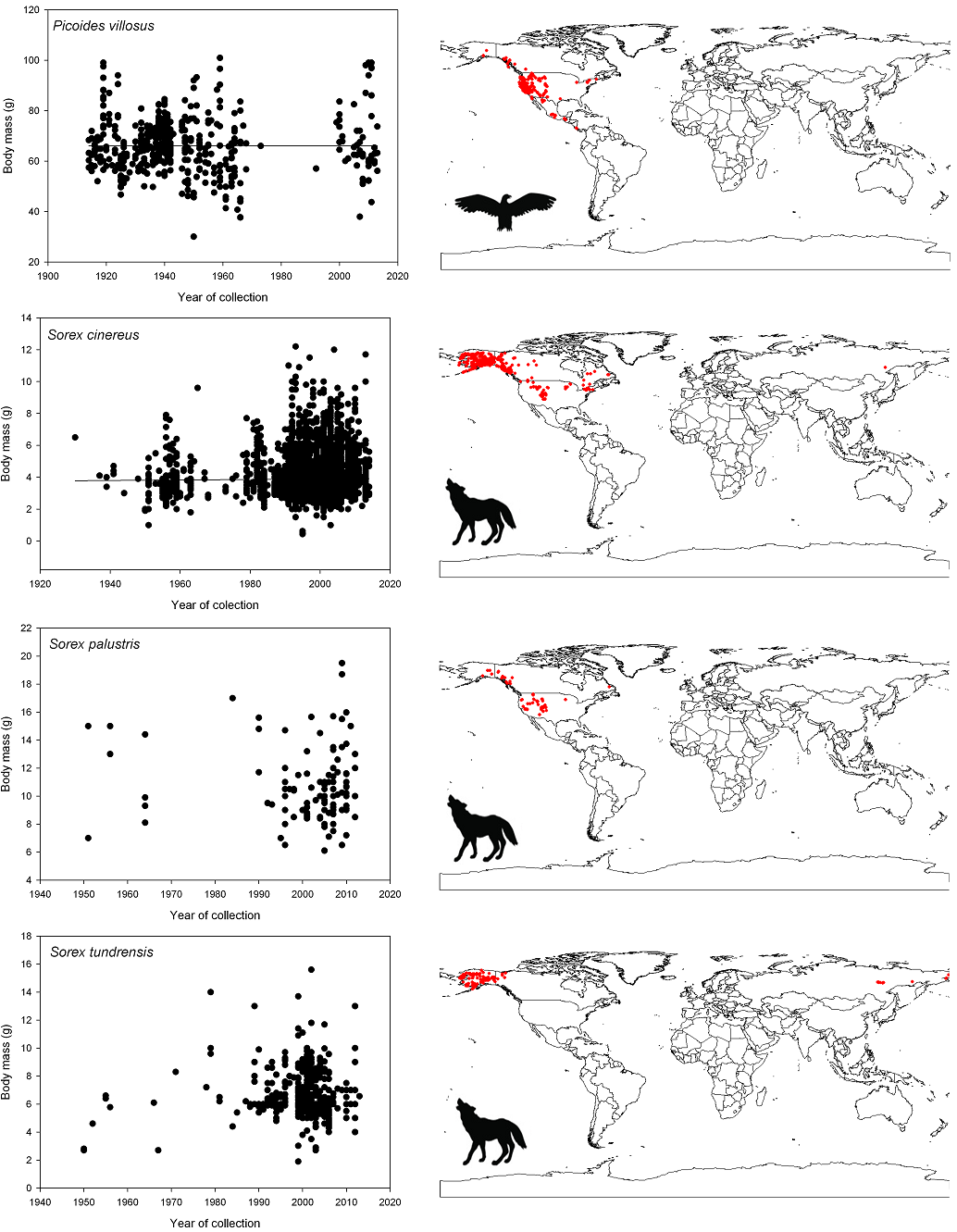
**

**Fig B (cont).**

**
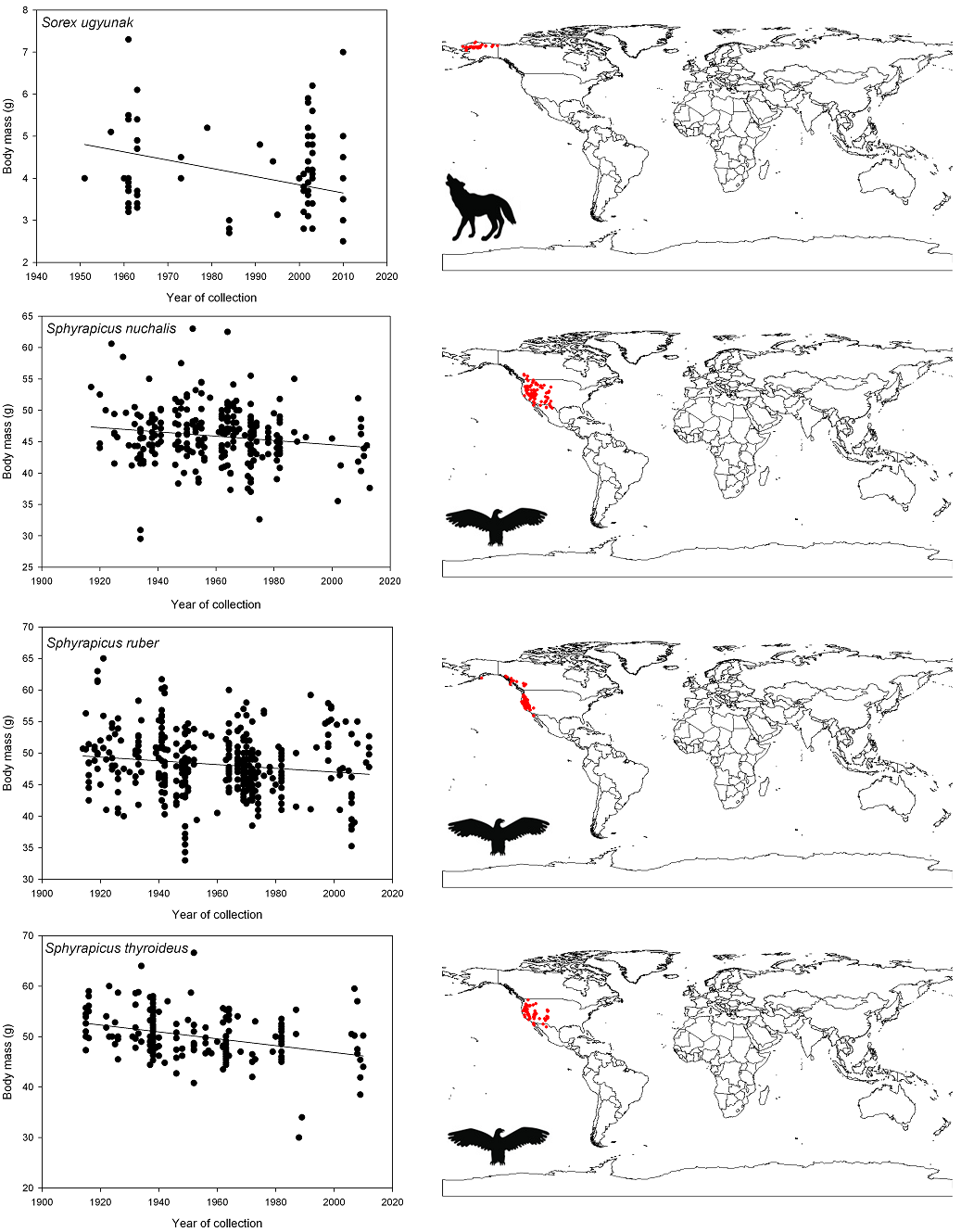
**

**Fig B (cont).**

**
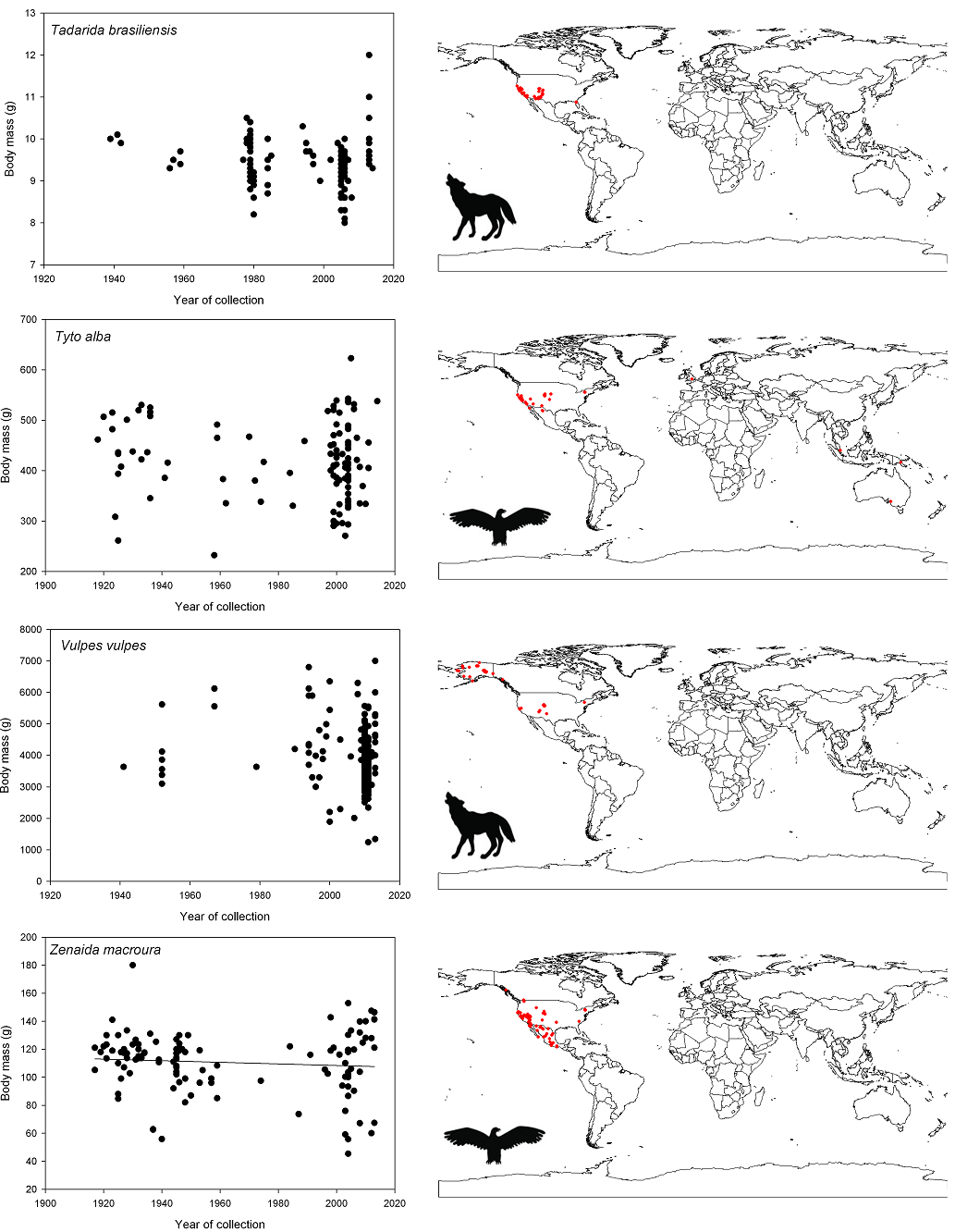
**

**Figure C.** Phylogenetic tree used to estimate the magnitude of the phylogenetic signal in r_year_. Aquatic species are shown in blue and terrestrial species are shown in green.

**Figure D.** World map showing the sites of collection for: *(a)* aquatic birds (sky blue) and mammals (dark blue), and *(b)* terrestrial birds (light green) and mammals (dark green).


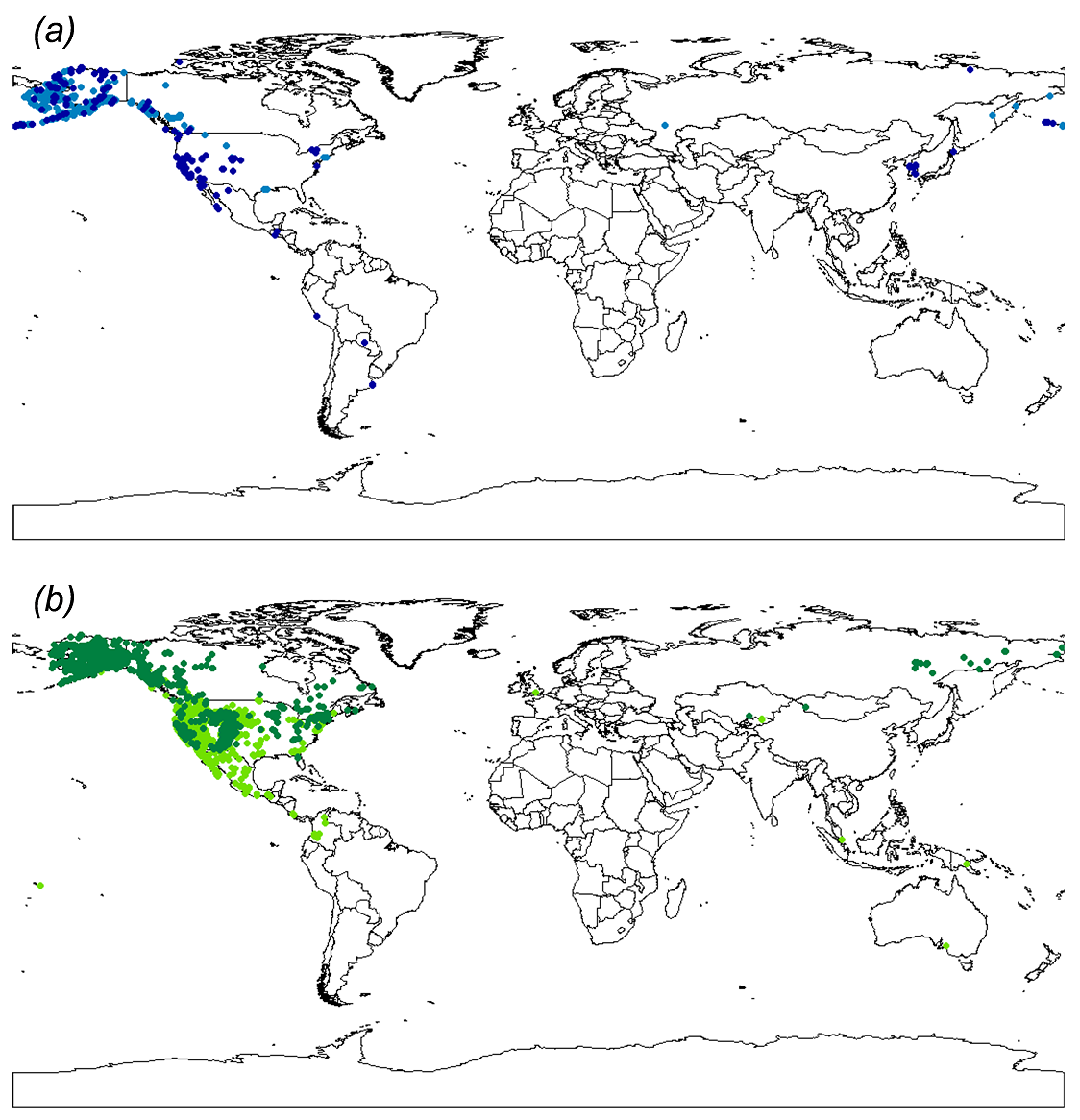

Supplement: S1 Appendix — (DOCX) [file pone.0183051.s001.docx]
